# Complete chloroplast genome sequence of ‘Field Muskmelon,’ an invasive weed to China

**DOI:** 10.1080/23802359.2021.1994888

**Published:** 2021-11-11

**Authors:** Dongsheng Bai, Xuegang Luo, Yonggang Yang

**Affiliations:** a School of Environment and Resource, Southwest University of Science and Technology, Mianyang, China; b College of Environment & Resource Science, Shanxi University, Taiyuan, China; c Shanxi Resources and Environment Survey of Coal Geology, Taiyuan, China

**Keywords:** *Cucumis*, Chloroplast genome, Phylogenetic analysis

## Abstract

*Cucumis melo* L. var. *agrestis* Naud., commonly known as ‘Field Muskmelon,’ is an annual invasive weed in many parts of China. However, there is very little available information about the chloroplast genome of this species. Here, we first report and characterize its complete chloroplast genome sequence based on Illumina paired-end sequencing data. The complete plastid genome was 155,402 bp, which contained inverted repeats (IR) of 25,514 bp separated by a large single-copy (LSC) and a small single copy (SSC) of 86,287 bp and 18,087 bp, respectively. The complete chloroplast genome contains 133 genes, comprising 87 protein-coding genes, 37 tRNA genes, 8 rRNA genes and 1 pseudogene. The overall GC content of the plastome is 36.9%. The phylogenetic analysis of 16 selected chloroplast genomes demonstrated that *C. melo* var. *agrestis* Naud was close to congeneric species *C. xhytivus*.

*Cucumis melo* L. var. *agrestis* Naud., is a trailing-vine weed which belongs to the family Cucurbitaceae. It is native to Africa and has invaded North and East China, such as Shandong, Hebei, Shanxi, Jiangsu, Anhui and Shanghai (Kerje and Grum 2000). This species is a common intruder in natural open areas, slopes, grasslands, wastelands, and fields. It can also infest crops, such as soybean, peanuts, and corn (Sohrabikertabad et al. 2013). However, there is very little available information about the chloroplast genome of this species. In plants, chloroplast (cp) genome provided useful information in systematics and biodiversity protection. It is thus urgent to take effective measures to protect this invasive species. Herein, we first reported and characterized its complete plastome based on Illumina paired-end sequencing data, which will contribute to the further studies on its genetic research and resource utilization. The annotated cp genome of *C. melo* has been deposited into GenBank with the accession number MT622320.

In this study, *C. melo* var. *agrestis* Naud was sampled from Taiyuan of Shanxi province, located at 112°43′13″E, 37°28′58″N. A voucher specimen (y.-g. Yang et al. S1451) was deposited in the Molecular Ecology Laboratory (MEL), College of Environment & Resource Science, Shanxi University (Taiyuan, Shanxi, China). The total genome DNA was extracted using the protocol (Zhang et al. 2019) and sent to Genewiz (https://www.genewiz.com.cn/, China) for next-generation sequencing using Illumina Hiseq Xten. Approximately 2 Gb high quality, 2 × 150 bp pair-end reads were obtained from High-throughput sequencing. The chloroplast genome of *Cucumis xhytivus* (Genbank accession no. KU821703) was used as a reference to exclude nuclear and chloroplast reads by Geneious Prime 2020.2 (Kearse et al. 2012). Filtered chloroplast reads were exploited for de novo assembly by the program NOVOPlasty (Dierckxsens et al. 2017) and direct-viewing in Geneious R11 (Biomatters Ltd., Auckland, New Zealand). Annotation was performed with the program Plann (Huang and Cronk 2015) and Sequin (http://www.ncbi.nlm.nih.gov/).

The chloroplast genome of *C. melo* var. *agrestis* Naud is a typical quadripartite structure with a length of 155,402 bp, which contained inverted repeats (IR) of 25,514 bp separated by a large single-copy (LSC) and a small single copy (SSC) of 86,287 bp and 18,087 bp, respectively. The cpDNA contains 133 genes, comprising 87 protein-coding genes, 37 tRNA genes, 8 rRNA genes and 1 pseudogene. Among the annotated genes, 13 of them contain one intron (*atp*F, *ndh*A, *ndh*B, *rps*16, *rpoC*1, *pet*B, *rpl*2, *trn*A-UGC, *trn*E-UUC, *trn*G-TCC, *trn*K-UUU, *trn*L-UAA and *trn*V-UAC), and three genes (*clp*P, *rps*12 and *ycf*3) contain two introns. The overall GC content of the plastome is 36.9%.

To identify the phylogenetic position of *C. melo* var. *agrestis* Naud, phylogenetic analysis was performed based on complete cp genomes from 15 Cucurbitaceae species with *Cucurbita maxima* as the outgroup species. All of these 16 complete cp genomes were aligned by the MAFFT version 7 software (Katoh and Standley 2013). Bayesian inference analysis was carried out in MrBayes v.3.2.2 (Ronquist et al. 2012) based on the GTR substitution model, which was selected by the Akaike Information Criterion (Posada and Buckley 2004) as implemented in the program Modeltest v.3.7 (Posada and Crandall 1998). The result strongly supported that *C. melo* var. *agrestis* is related to the congeneric *C. xhytivus* (Figure 1). Our findings can be further used for population genomic and phylogenomic studies of Cucurbitaceae. It will also provide fundamental data for the protective, utilization and management of this invasive species.

**Figure 1.**
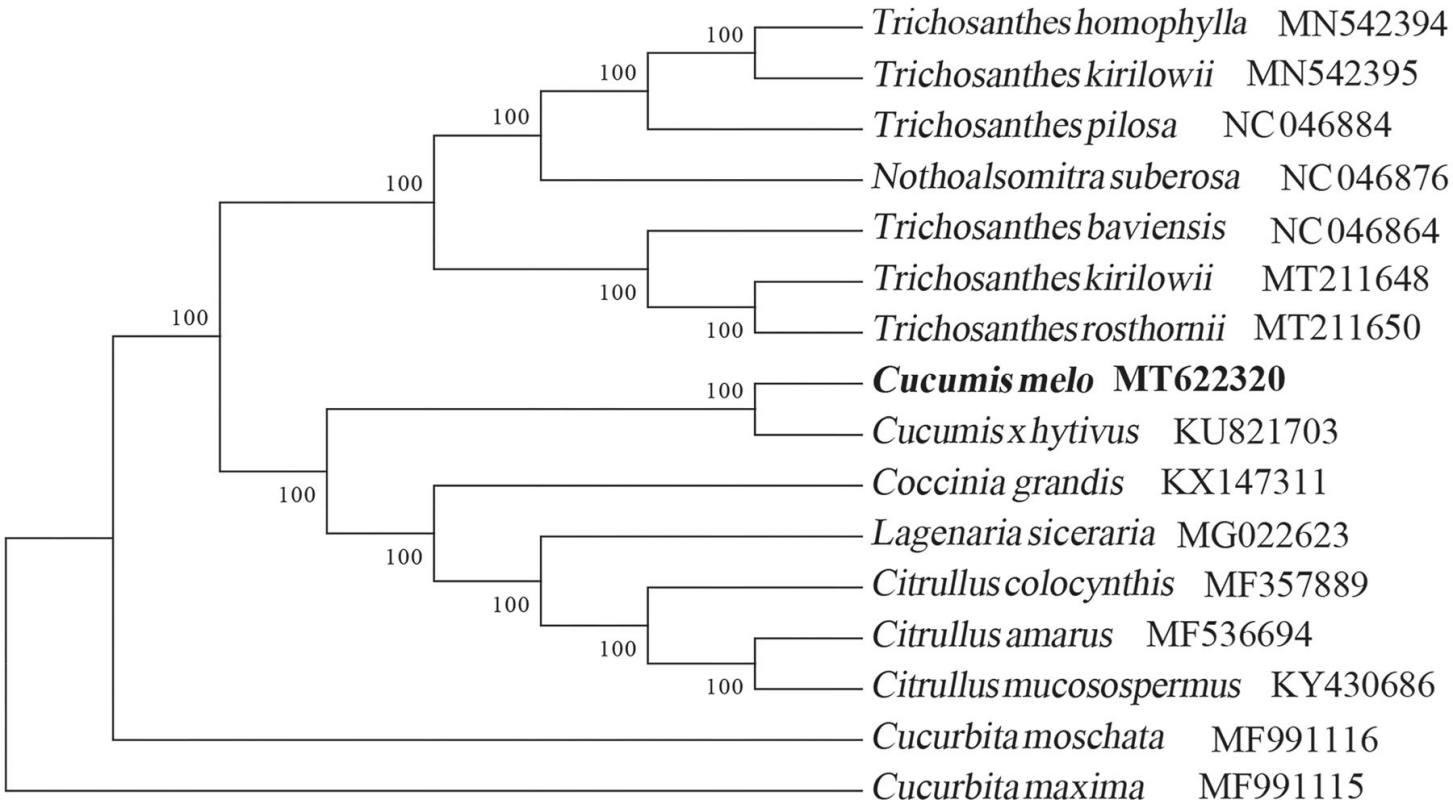
Bayesian inference analysis based on 16 complete chloroplast genomes with Cucurbita maxima as an outgroup. The bootstrap support values (>90%) were shown above the branches. The position of C. melo var. agrestis Naud was marked with a bold and GenBank accession numbers were listed behind each species name.

## Data Availability

The data that support the findings of this study are openly available in GenBank of NCBI at (https://www.ncbi.nlm.nih.gov/nuccore/MT622320) under the accession no. MT622320.
